# The incidence trends of liver cirrhosis caused by nonalcoholic steatohepatitis via the GBD study 2017

**DOI:** 10.1038/s41598-021-84577-z

**Published:** 2021-03-04

**Authors:** Mimi Zhai, Zhide Liu, Jianhai Long, Qingxiang Zhou, Leping Yang, Qin Zhou, Sushun Liu, Yu Dai

**Affiliations:** 1 Xiangya Nursing School, Central South University, Changsha, 410013 Hunan China; 2 Department of General Surgery, The Second Xiangya Hospital, Central South University, Changsha, 410011 Hunan China; 3 Department of General Surgery, The Central Hospital of Shaoyang, Shaoyang, 42200 Hunan China; 4 Department of Respiratory, Beijing Tiantan Hospital, Capital Medicine University, Beijing, 100050 China; 5 Clinical Nursing Teaching and Research Section, Department of Orthopedics, The Second Xiangya Hospital, Central South University, Changsha, 410011 Hunan China

**Keywords:** Liver diseases, Liver cirrhosis, Non-alcoholic steatohepatitis

## Abstract

Nonalcoholic steatohepatitis (NASH) has rapidly become the most common cause of chronic liver diseases. We aimed to explore the incidence and distribution characteristics of NASH by sex, region and sociodemographic index (SDI). We collected data, including sex and region, on NASH-related liver cirrhosis from the 2017 GBD study. The age-standardized incidence rates (ASRs) and estimated annual percentage changes (EAPCs) were used to estimate the incidence trend and distribution characteristics. Globally, the incidence of liver cirrhosis caused by NASH increased from 178,430 cases in 1990 to 367,780 cases in 2017, an increase of approximately 105.56%. The ASR of NASH increased by an average of 1.35% per year (95% CI 1.28–1.42). Meanwhile, large differences in the ASR and the EAPC were observed across regions. The middle-high SDI region had the highest increase among all five SDI regions, followed by middle SDI region. In addition, Eastern Europe, Andean Latin America and Central Asia showed a more significant growth trend of ASR. In contrast, the high SDI region demonstrated the slowest increasing trend of ASR, and the high-income Asia Pacific demonstrated a decreasing trend among the 21 regions. Liver cirrhosis has caused a huge and rising health burden in many countries and regions. In addition, with the growth of obesity, population and aging, NASH might replace viral hepatitis as the most important cause of liver cirrhosis in the near future. Therefore, appropriate interventions are needed in coming decades to realize early diagnosis and prevention of NASH-related liver cirrhosis.

## Introduction

Liver cirrhosis is an important process in liver cancer progression, accounting for more than one million deaths per year throughout the world^1–3^. Recent studies have shown that the transition from liver cirrhosis to cancer is a dynamic process regulated by the occurrence of clinical decompensation events^4,5^. Thus, the corresponding preventive strategies and treatments should be formulated according to the clinical stages of liver cirrhosis^6–9^.

Nonalcoholic steatohepatitis (NASH) is defined as a disease with > 5% hepatic steatosis and inflammation with or without fibrosis^10^. NASH is considered a common cause of liver cirrhosis, which is due to remarkable changes in lifestyle and geographic disparities around the world^11,12^. Approximately 20–50% of NASH patients may suffer from cirrhosis within 10 years^13–15^. Over the past three decades, the incidence of liver cirrhosis caused by NASH has varied dramatically among countries, and a total of 90 million people have suffered from NASH. All these results indicated that a healthy lifestyle and appropriate interventions are needed in the coming decades^8^.

The Global Burden of Disease (GBD) study reports the burden of liver cirrhosis caused by NASH based on sex, region, and sociodemographic index (SDI) from 1990 to 2017 in 195 countries and territories. By analyzing the latest GBD data, researchers can establish more prudent prevention strategies. By using data from the GBD study, several previous studies have assessed the incidence, prevalence and mortality of liver cirrhosis and liver cancer^1,16,17^. Few studies have been concerned with the trends in the incidence of liver cirrhosis caused by NASH around the world. In this study, we presented the incidence trends of liver cirrhosis caused by NASH by sex, region and SDI.

## Results

### Incidence of liver cirrhosis caused by NASH

**Figure 1:**
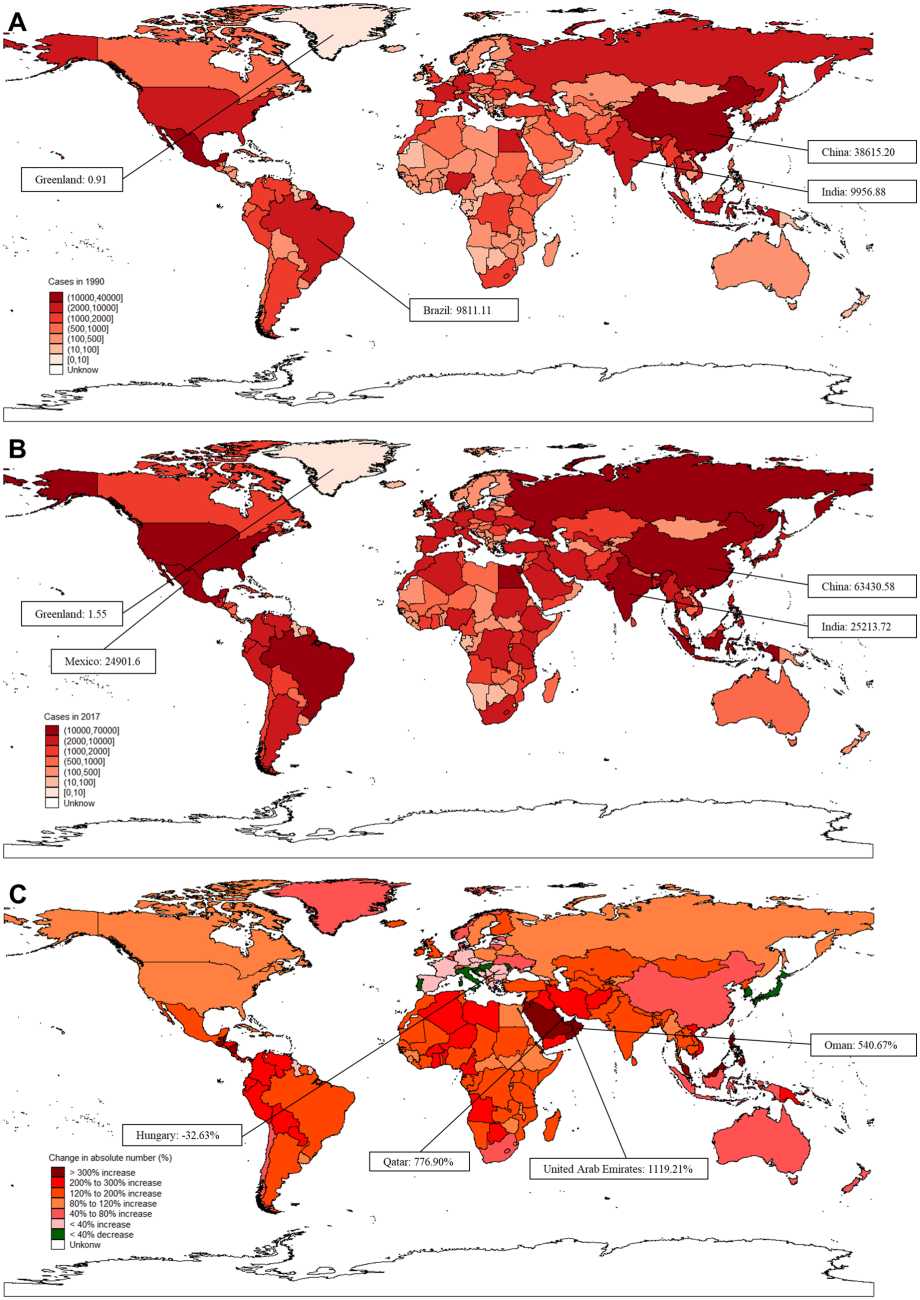
The overall incident cases of liver cirrhosis caused by NASH in 195 countries and territories. (**A**) The incident cases in 1990 across the world. (**B**) The incident cases in 2017 across the world. (**C**) The change in incident cases of liver cirrhosis caused by NASH from 1990 to 2017 across the world. Figures were created by STATA/MP 13.1 software. The SHP data of the world map were converted to STATA data via the “shp2dta” command, and maps were drawn via the “spmap” command after incorporating variables.

The global incidence of liver cirrhosis caused by NASH increased 106.12% from 1990 to 2017. The countries with the highest incident cases in the world were China (38.62 × 10^3^), India (9.96 × 10^3^) and Brazil (9.81 × 10^3^), which accounted for approximately 32.68% of the total cases of NASH-related liver cirrhosis (Fig. 1A–C, Table 1). Although China had the highest number of incident cases of liver cirrhosis caused by NASH, its growth rate was lower than the global average level (Fig. 1C, Supplementary Table S1). Moreover, 35 countries out of 195 countries accounted for 80.25% of global incident cases. A total of 129 countries and territories, most of which were developing countries, experienced a prominent increase of more than 100%. Among all countries, the highest growth rate of incident cases was found in the United Arab Emirates (1119.21%), followed by Qatar (776.90%) and Oman (540.67%) (Supplementary Table S1). In contrast, an average 20.1% decrease in incident cases was found in 8 of 195 countries and territories, and the most significant decrease was observed in Hungary (− 32.63%), followed by Moldova (− 25.89%), South Korea (− 24.03%), Croatia (− 22.70%), Portugal (− 20.48%), Italy (− 13.44%), Slovenia (− 10.74%), and Japan (− 10.60%) (Fig. 1B).

**Table 1:** The incident cases, age-standardized incidence, and temporal trend of liver cirrhosis caused by NASH between 1990 and 2017.

Characteristics	1990		2017		1990–2017
	Incident cases No. × 10^3^ (95% UI)	ASR per 100,000 No. (95% UI)	Incident cases No. × 10^3^ (95% UI)	ASR per 100,000 No. (95% UI)	EAPC No. (95% CI)
Overall	178.43 (162.93–195.85)	3.31 (3.02–3.63)	367.78 (334.46–403.73)	4.81 (4.38–5.28)	1.35 (1.28–1.42)
**Sex**					
Male	106.21 (96.99–116.23)	3.91 (3.57–4.28)	212.33 (193.93–232.72)	5.54 (5.06–6.07)	1.24 (1.17–1.32)
Female	72.22 (65.62–79.58)	2.70 (2.45–2.97)	155.45 (140.60–170.78)	4.08 (3.69–4.49)	1.51 (1.44–1.57)
**Socio-demographic index**					
Low	14.25 (12.92–15.76)	2.04 (1.85–2.26)	35.04 (31.29–39.28)	2.72 (2.43–3.05)	1.03 (0.90–1.16)
Low-middle	31.41 (28.34–34.91)	3.01 (2.71–3.34)	74.40 (66.69–82.84)	4.36 (3.91–4.86)	1.36 (1.34–1.37)
Middle	56.49 (51.87–61.46)	3.64 (3.34–3.96)	124.91 (114.34–136.16)	5.98 (5.47–6.51)	1.76 (1.69–1.84)
Middle-high	42.82 (39.47–46.69)	3.85 (3.55–4.20)	85.13 (77.73–92.99)	6.14 (5.60–6.70)	1.77 (1.65–1.89)
High	32.64 (28.92–37.10)	3.38 (2.99–3.84)	46.92 (42.79–51.91)	4.12 (3.75–4.55)	0.62 (0.57–0.68)
**Region**					
Western Sub-Saharan Africa	6.38 (5.70–7.15)	3.32 (2.96–3.72)	16.36 (14.45–18.57)	3.77 (3.33–4.28)	0.36 (0.31–0.40)
Southern Sub-Saharan Africa	1.60 (1.44–1.78)	3.05 (2.75–3.39)	2.79 (2.49–3.14)	3.61 (3.21–4.06)	0.54 (0.44–0.63)
Eastern Sub-Saharan Africa	6.58 (5.95–7.29)	3.44 (3.11–3.81)	15.16 (13.53–16.97)	3.85 (3.44–4.32)	0.32 (0.22–0.42)
Central Sub-Saharan Africa	1.50 (1.33–1.69)	2.73 (2.41–3.08)	3.95 (3.43–4.61)	3.25 (2.82–3.79)	0.59 (0.50–0.69)
Oceania	0.14 (0.12–0.17)	2.19 (1.86–2.56)	0.39 (0.33–0.46)	3.11 (2.60–3.69)	1.28 (1.24–1.32)
High-income North America	7.00 (6.57–7.45)	2.49 (2.34–2.65)	14.09 (13.04–15.15)	3.91 (3.61–4.20)	1.62 (1.57–1.68)
North Africa and Middle East	15.44 (13.57–17.55)	4.53 (3.98–5.15)	45.31 (39.61–51.41)	7.55 (6.60–8.57)	1.99 (1.88–2.10)
Tropical Latin America	9.98 (9.21–10.84)	6.51 (6.00–7.06)	22.69 (20.80–24.60)	10.37 (9.51–11.25)	1.72 (1.67–1.77)
Southern Latin America	2.19 (1.95–2.44)	4.41 (3.93–4.93)	4.23 (3.77–4.72)	6.45 (5.74–7.19)	1.39 (1.38–1.41)
Central Latin America	15.35 (13.80–17.01)	9.35 (8.41–10.36)	40.51 (36.42–44.71)	15.86 (14.26–17.50)	1.95 (1.91–1.98)
Andean Latin America	2.34 (2.09–2.61)	6.11 (5.45–6.81)	7.96 (7.16–8.83)	12.96 (11.65–14.37)	2.94 (2.85–3.04)
Western Europe	14.94 (12.77–17.46)	3.87 (3.31–4.53)	18.95 (16.80–21.73)	4.38 (3.88–5.02)	0.27 (0.19–0.35)
Eastern Europe	8.69 (8.10–9.35)	3.83 (3.57–4.12)	15.96 (14.85–17.20)	7.59 (7.07–8.18)	3.26 (2.99–3.54)
Central Europe	5.83 (5.26–6.48)	4.70 (4.24–5.22)	7.17 (6.51–7.90)	6.25 (5.67–6.88)	1.32 (1.19–1.44)
Caribbean	2.18 (1.98–2.42)	6.19 (5.60–6.86)	4.49 (4.01–4.99)	9.69 (8.68–10.79)	1.55 (1.49–1.60)
Australasia	0.48 (0.42–0.54)	2.35 (2.05–2.67)	0.81 (0.72–0.91)	2.87 (2.55–3.20)	0.81 (0.67–0.94)
Southeast Asia	15.37 (14.03–16.82)	3.29 (3.01–3.60)	36.23 (32.82–39.99)	5.49 (4.97–6.06)	1.86 (1.80–1.92)
South Asia	13.25 (12.23–14.36)	1.19 (1.10–1.29)	33.10 (30.41–36.09)	1.86 (1.71–2.02)	1.57 (1.44–1.70)
East Asia	40.79 (37.21–44.78)	3.24 (2.96–3.56)	66.99 (60.79–73.22)	4.51 (4.09–4.93)	0.99 (0.76–1.22)
Central Asia	2.35 (2.14–2.57)	3.36 (3.07–3.69)	5.54 (5.01–6.13)	6.10 (5.51–6.75)	2.44 (2.32–2.57)
High-income Asia Pacific	6.06 (4.98–7.33)	3.49 (2.87–4.22)	5.06 (4.31–5.96)	2.71 (2.30–3.18)	− 1.22 (− 1.36–1.08)

In this period, liver cirrhosis caused by NASH was more common in men. However, the ratio of males to females slightly decreased from 1.47:1 to 1.37:1 during the study period (Table 1). The incidence increased to different degrees, for example, in all five SDI regions and in both sexes. In all 21 geographical regions, only in the high-income Asia Pacific region did the incidence decrease (by 16.5%); this finding was in stark contrast to those of the remaining regions. The corresponding maximum number of incident cases was found in the middle SDI region (124.91 × 10^3^), males (212.33 × 10^3^) and East Asia (66.99 × 10^3^). However, the low SDI region and low-middle SDI region had a relatively high growth rate of incident cases among the five SDI regions.

### ASR and its trend of liver cirrhosis caused by NASH

**Figure 2:**
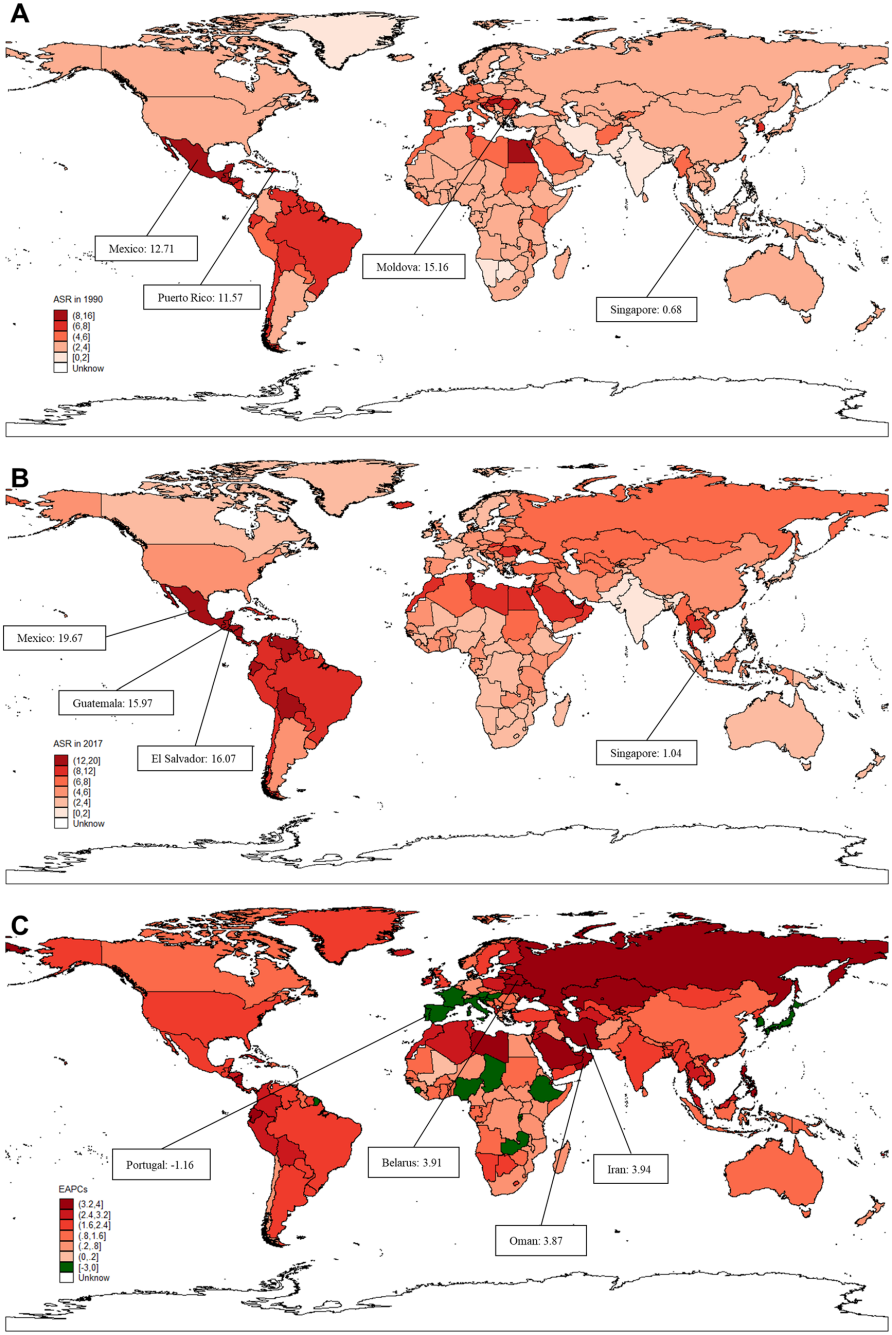
The global burden of liver cirrhosis incidence caused by NASH in 195 countries and territories. (**A**) The ASR of liver cirrhosis incidence caused by NASH in 1990. (**B**) The ASR of liver cirrhosis incidence caused by NASH in 2017. (**C**) The ASR trend of liver cirrhosis caused by NASH from 1990 to 2017 across the world. Figures were created via the method mentioned in Fig. 1.

The age-standardized incidence rates (ASRs) and estimated annual percentage changes (EAPCs) of liver cirrhosis caused by NASH varied considerably. Globally, the ASR increased with an annual average of 1.35% per year (95% CI 1.28–1.42), from 3.31 (95% CI 3.02–3.63) per 1,000,000 to 4.81 (95% CI 4.38–5.28) per 1,000,000 (Fig. 2A,B, Table 1). The highest ASR was found in Mexico in 2017, with a value of 19.67 per 1,000,000, followed by El Salvador and Guatemala (Fig. 2B). Additionally, ASR displayed a significant increase in NASH-related liver cirrhosis, and the most remarkable increase was found in the middle-high SDI region, with an EAPC of 1.77 (95% CI 1.65–1.89), followed by the middle SDI region (Fig. 2C). Geographically, the ASR of liver cirrhosis caused by NASH presented an increasing trend in 20 regions from 1990 to 2017, except for the high-income Asia Pacific region (Fig. 2C, Table 1). In parallel, the highest ASR was noted in Central Latin America, and the greatest increase in ASR was observed in Eastern Europe, with an EAPC value of 3.26 (95% CI 2.99–3.54). Regarding sex, the ASR showed a higher growing trend globally in females than in males from 1990 to 2017 (Table 1). Among the 195 countries and territories, 177 countries and territories showed an increasing trend in the ASR of liver cirrhosis caused by NASH. Moreover, 113 countries and territories demonstrated faster growth of ASR than the global average (Supplementary Table S1). In contrast, 14 countries demonstrated a decreasing trend of ASR, including South Korea, Portugal, Hungary, Italy, Burundi, Spain, Japan, France, Austria, Nigeria, Slovenia, Zambia, Rwanda and Croatia (Supplementary Table S1). In addition, the ASR of liver cirrhosis in Luxembourg, Sierra Leone, Chad and Ethiopia remained stable during the study period (Fig. 2A,B). The maximum value of ASR was found in Mexico (19.67 per 1,000,000), followed by El Salvador (16.07 per 1,000,000) and Guatemala (15.97 per 1,000,000). In addition, the countries with the most obvious increasing trend of ASR in liver cirrhosis caused by NASH were Iran (3.94; 95% CI 3.71–4.18), Belarus (3.91; 95% CI 3.55–4.26) and Oman (3.87; 95% CI 3.39–4.35) (Fig. 2B,C). Notably, all Sub-Saharan African regions demonstrated a minor increase in ASR during the study period, similar to some of the developed countries (Table 1, Supplementary Table S1).

**Figure 3:**
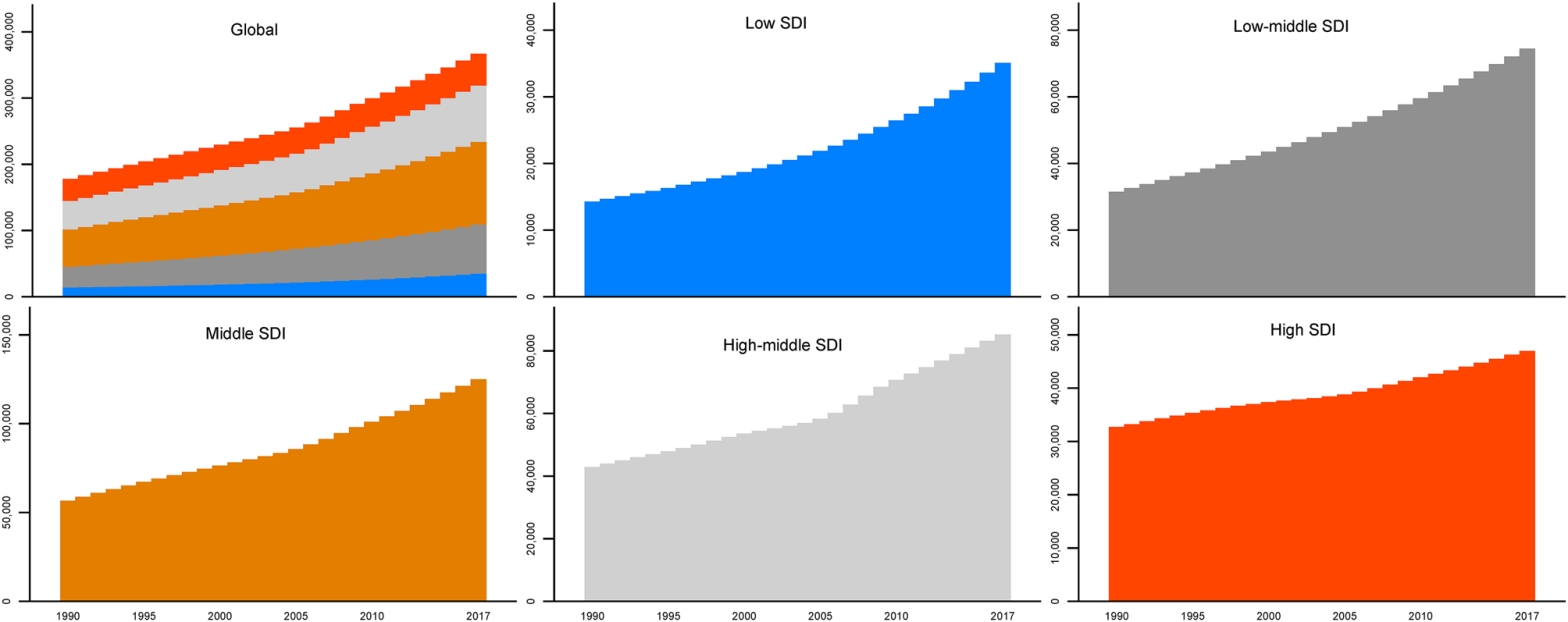
Liver cirrhosis incident cases caused by NASH and SDI regions from 1990 to 2017. Figures were created by STATA/MP 13.1 software. The bar charts were drawn via the “graph bar” command, and the cumulative bar charts were drawn by adding the option “stack”.

A gradient upward trend of incident cases was observed across the five SDI regions instead of a continuous growth trend (Fig. 3). The incident cases most significantly increased in the middle SDI region, followed by the high-middle SDI region. Additionally, the incidence of liver cirrhosis caused by NASH only increased 43.75% during the study period in the high SDI region, which was the region with the lowest increase in the number of cases among the five SDI regions (Fig. 3).

## Discussion

Because of progressive fibrosis, NASH is inextricably linked to a significant long-term outcome, and a few NASH patients may even die of various liver-related complications^18^. Although the number of newly diagnosed cases increased, the upward trend in the incidence of NASH-related liver cirrhosis varied in different regions. This research analyzed the trends in the incidence of liver cirrhosis caused by NASH globally, contributing to optimizing the allocation of health resources and improving health outcomes for patients suffering NASH-related liver cirrhosis.

Obesity and metabolic syndrome are considered the main risk factors for NASH-related cirrhosis. Previous studies found that the prevalence of nonalcoholic fatty liver disease (NAFLD) was increasing at the same rates as obesity^19^. Moreover, obesity was more common in developed countries. However, the gap in obesity prevalence between developed and developing countries is narrowing^20^. Compared to Western countries such as America and the United Kingdom, the countries in the Arabian Peninsula and Asian countries, such as the United Arab Emirates and India, also have high levels of obesity and metabolic syndrome^20–22^. Our study also found that the incidence was evolving in Asia and Eastern Europe. This might be related to a sedentary lifestyle and overnutrition. High-fat foods, lack of exercise, and diabetes might affect lipid metabolism and induce NASH-related liver cirrhosis. Moreover, the process of NASH-related liver cirrhosis might be accelerated. Additionally, the westernized lifestyle might also be responsible for the increasing trend of NASH-related cirrhosis in these areas^23^. Meanwhile, overweight and obesity among children and adolescents also lead to a significant increase in the burden of liver cirrhosis caused by NASH^24–27^. The increase in NASH-related cirrhosis is partly caused by obesity, but some areas with low body mass index still have an increasing incidence, indicating that there are other cofactors contributing to the onset and progression of cirrhosis in NASH patients^28^. Lean NASH might also contribute to the rising trend of liver cirrhosis caused by NASH. Studies have found that there may be a large number of nonobese people in developing countries, such as China, India and Mexico, suffering from NASH-induced cirrhosis^29–31^. Patients with lean NASH have been shown to have higher mortality and shorter survival times after liver transplantation^32^. With the increase in aging populations and the life expectancy index, the possibility of fibrotic response formation progressively increases, causing a serious public health problem^33^. Thus, more effective and targeted strategies should be formulated to reverse the rising trend.

In our study, the incidence of eight European countries decreased during the period. In accordance with a previous global survey, it was found that the upward trend of obesity has been effectively controlled in northwestern Europe and the high-income Asia Pacific region during the last two decades^20^. Moreover, regional changes in BMI in eastern European girls did not show obvious differences^9,20,34^. These observations might help explain why the incidence decreased in the eight European countries, which were different from all other countries and territories. Additionally, the Mediterranean diet in Italy and seven other Mediterranean countries could also help to reduce the risk of obesity, diabetes and metabolic syndrome^35^. Our study also demonstrated that the trend in ASR of NASH-related cirrhosis was increasing slowly in Sub-Saharan African regions. Previous studies found that race and ethnicity were both risk factors for NASH^36,37^. Compared with Hispanics and non-Hispanic white individuals, African Americans obtained the lowest prevalence of NASH^37^. In addition, familial clustering of NASH might also affect the trend of NASH-related cirrhosis. Studies also indicated that rs738409G, a genetic marker that was related to NASH, had higher proportions in Hispanics and lower proportions in African Americans^38^.

Our study also demonstrated that the ASR increased during our study period, especially in the high-middle and middle SDI regions. In a previous study, researchers found that the yearly cumulative incidence of hepatocellular carcinoma was 2.6% per year in patients with liver cirrhosis caused by NASH^39^. With the development of diagnostic technology and an understanding of NASH, the prevalence has gradually increased in recent years^40^. In addition to the improvement of medical care, population growth may also be one of the reasons for the rising trend of NASH-related liver cirrhosis. Moreover, immigration might also lead to a rising trend^41^. Different from local residents, the diet and living habits of immigrants might be different, which might be related to the incidence. Previous studies indicated that the prevalence of NASH and NASH-related cirrhosis increased with age^36,42^. Our study also found that the incidence was higher in middle-aged individuals, especially in women. This might be related to the high incidence of metabolic conditions in these individuals, as well as severe cirrhosis and liver cancer^43^.

Additionally, middle-aged females were more likely to suffer from NASH-related cirrhosis in our study than males. Yang et al. observed that postmenopausal women were more vulnerable to serious liver cirrhosis than premenopausal women in a cross-sectional study^44^. The difference in incidence by sex may be caused by sex hormones in liver cirrhosis caused by NASH. Additionally, the higher increasing trend of ASR in NASH-related liver cirrhosis in females might be driven by a higher prevalence of obesity and lower consumption of alcohol^45,46^. Therefore, preventive measures that are more targeted at women should be established in recent years.

Although China has the largest number of incident cases of liver cirrhosis caused by NASH, the amplitude of ASR variation is lower than the world average level. Unlike Western countries, Asian individuals, such as China and Japan, have a higher prevalence of NASH leading to cirrhosis^23^. In contrast, our research showed a relatively lower growth rate of the incidence of liver cirrhosis caused by NASH in China than in Western countries. China suffers from fewer obesity and metabolic conditions than Western countries^20^. However, China has also faced a greater obesity problem in recent years than before. Thus, raising investment in effective interventions should be introduced all around the world, especially in Asian countries^47^. In 2016, a strategy in China, called ‘Health China 2030’, was set to address new challenges, including NASH-related cirrhosis, for the maintenance of national health. Similar policies should be extended to other countries in the long term.

Although this study has provided further information for supporting medical decisions, there are some limitations of our study. GBD study lacks data in remote villages or some countries. This might affect the estimated trend of NASH-related cirrhosis. In addition, the GBD data of liver cirrhosis did not include the data of risk factors, such as BMI, diabetes, and blood lipids. Therefore, we could not further analyze the risk factors for NASH-related cirrhosis. Moreover, it was impossible to explore the relationship between BMI and NASH by using GBD data. Finally, due to a lack of relevant data, such as BMI and blood lipids, we could not study the trend of lean NASH in GBD study. Now the GBD study has been updated to GBD study 2019. In our study, we used the date from the GBD study 2017. The item B.4.1.4 of liver cirrhosis was changed from cirrhosis due to NASH in 2017 to cirrhosis and other chronic liver diseases due to NAFLD in 2019. As the definition of NASH changed, the number of patients and trends also changed dramatically. According to the previous definition of cirrhosis due to NASH in the GBD study 2017, the relevant data have not been updated in the new version of the GBD study 2019. Moreover, the study used data on cirrhosis and other chronic liver diseases due to NAFLD from the GBD study 2019, which might be completely different from that used the data on liver cirrhosis due to NASH from the GBD study 2017. Thus, this study was not suitable for updating with data from the GBD study 2019, and we will use the new data of GBD study 2019 in our further studies.

In conclusion, NASH has emerged as the most common cause of chronic liver disease worldwide^11,28^. Due to a lack of awareness of NASH and missed diagnosis, the actual incidence of NASH-related cirrhosis might be underestimated. Therefore, it is of great significance to make liver cirrhosis caused by NASH an urgent public health priority worldwide. More targeted medical and public measures should be initiated and implemented immediately.

## Materials and methods

### Study data

Detailed information on liver cirrhosis caused by NASH from 1990 to 2017, available for exploration by an online visualization tool, was retrieved from the GBD 2017 study for 195 countries and territories. According to the SDI and geographical location, these areas were correspondingly divided into five regions (low, low-middle, middle, high-middle, and high SDI regions) and 21 regions. Details on the methods for the GBD 2017 have been clarified in prior studies^2,48^.

### Ethical committee

All date was available at http://ghdx.healthdata.org/gbd-2017. As a noncommercial user of IHME websites, we followed the Creative Commons Attribution-NonCommercial-NoDerivatives 4.0 International License and Section 7 of the University of Washington’s Website Terms and Conditions of Use in our study. In our study, we used, modified and built the IHME data via the Creative Commons Attribution-NonCommercial-NoDerivatives 4.0 International License. The University of Washington IRB Committee approved the Global Burden of Diseases, Injuries, and Risk Factors study, STUDY00009060. The study is approved until 2 December 2021.

### Statistical analysis

For the purpose of describing and evaluating the landscape of liver cirrhosis caused by NASH incidence from 1990 to 2017, ASR and EAPC were adopted in our study^48,49^. By using ASR, we could assess the incidence of NASH-related liver cirrhosis and establish targeted preventive strategies for liver cirrhosis caused by NASH^50^. EAPC was used to quantify the trends of ASR of liver cirrhosis caused by NASH among different populations in a period^51^. Both the EAPC value and the lower boundary of the 95% CI were greater than 0, indicating an increasing trend of ASR. In contrast, both the EAPC value and the upper boundary were less than 0, indicating a decreasing trend of ASR. When the 95% CI contained 0, it indicated a constant trend of ASR. Statistical analyses were performed by R software (R 3.5.1 software, Institute for Statistics and Mathematics) and STATA/MP (STATA 13.1, StataCorp LLC). Values of *p* < 0.05 were considered statistically significant.

## Supplementary Information

Supplementary Table S1.

## Abbreviations

NASH: Nonalcoholic steatohepatitis
GBD: Global burden of disease
SDI: Sociodemographic index
ASRs: Age-standardized incidence rates
EAPCs: Estimated annual percentage changes
CI: Confidence interval
NAFLD: Nonalcoholic fatty liver disease
