# Supplementary material for: The incidence trends of liver cirrhosis caused by nonalcoholic steatohepatitis via the GBD study 2017
Source: Sci Rep. 2021 Mar 4;11:5195. doi: 10.1038/s41598-021-84577-z (PMC7933440; doi:10.1038/s41598-021-84577-z)
Supplement: Supplementary file 1 — Supplementary Table S1. [file 41598_2021_84577_MOESM1_ESM.docx]

The incidence trends of liver cirrhosis caused by Non-alcoholic steatohepatitis via the GBD Study 2017

Mimi Zhai^1*^, Zhide Liu^2, 3*^, Jianhai Long^4^, Qingxiang Zhou^2^, Leping Yang^2^, Qin Zhou^2^, Sushun Liu^2*^, Yu Dai^5*^

^1^ Xiangya Nursing School, Central South University, Changsha, Hunan 410013, China

^2^ Department of General Surgery, the Second Xiangya Hospital, Central South University, Changsha, Hunan 410011, China

^3^ Department of General Surgery, the Central Hospital of Shaoyang, Shaoyang, Hunan 42200, China

^4^ Department of Respiratory, Beijing Tiantan Hospital, Capital Medicine University, Beijing 100050, China

^5^ Department of Orthopedics, the Second Xiangya Hospital, Central South University, Changsha, Hunan 410011, China

Mimi Zhai and Zhide Liu contributed equally to this article.

**Correspondence to**: Yu Dai, Department of Orthopedics, the Second Xiangya Hospital, Central South University, Changsha, Hunan 410011, China. [53450450@qq.com](mailto:53450450@qq.com) or daiyu@csu.edu.cn. Telephone: +86-731-85295120.

Sushun Liu, MD, Department of General Surgery, The Second Xiangya Hospital, Central South University, Changsha, Hunan 410011, China. surun9566@126.com or [surun9566@csu.edu.cn](mailto:surun9566@csu.edu.cn). Telephone: +86-731-85295120 Fax: +86-731-85533525

**Author contributions**

Study design: Dai Y and Zhai MM. Data collection: Zhai MM and Liu SS. Data analysis: Liu ZD and Liu SS. Figures: Liu SS Long JH and Zhou QX. Manuscript writing: Yang LP, Zhou Q and Zhai MM. Manuscript proofing: Zhai MM, Dai Y and Liu ZD.

**Supplemental Table 1. The incident cases and temporal trend of liver cirrhosis caused by NASH in 195 countries and territories**

| Characteristics | Case in 1990 | ASR in 1990 | Case in 2017 | ASR in 2017 | Change in absolute number (%) | EAPC (95% CI) |
| --- | --- | --- | --- | --- | --- | --- |
| Ethiopia | 1558.39 | 3.03 | 3124.4 | 3.04 | 100.49 | -0.07(-0.23-0.09) |
| Chad | 173.28 | 2.87 | 437.5 | 2.87 | 152.48 | -0.08(-0.18-0.03) |
| Sierra Leone | 155.49 | 4.11 | 330.8 | 4.22 | 112.75 | -0.09(-0.18-0.00) |
| Luxembourg | 23.41 | 6.04 | 38.42 | 6.51 | 64.12 | -0.11(-0.25-0.03) |
| Croatia | 317.82 | 6.49 | 245.66 | 5.75 | -22.7 | -0.15(-0.27--0.03) |
| Slovenia | 144.94 | 7.28 | 129.38 | 6.25 | -10.74 | -0.17(-0.33-0.00) |
| Zambia | 316.58 | 4 | 723.68 | 4.17 | 128.59 | -0.17(-0.33-0.00) |
| Rwanda | 318.65 | 4.39 | 538.59 | 4.29 | 69.02 | -0.17(-0.35-0.01) |
| Mali | 296.5 | 3.42 | 773.77 | 3.82 | 160.97 | 0.19(0.01-0.38) |
| Nigeria | 3174.95 | 3.54 | 7124.01 | 3.46 | 124.38 | -0.23(-0.36--0.11) |
| South Sudan | 230.03 | 3.91 | 426.92 | 4.29 | 85.59 | 0.24(0.15-0.34) |
| Malawi | 402.48 | 4.16 | 808.12 | 4.7 | 100.79 | 0.24(0.16-0.32) |
| South Africa | 1247.12 | 3.39 | 2079.46 | 3.78 | 66.74 | 0.28(0.16-0.39) |
| Afghanistan | 507.73 | 5.07 | 1572.34 | 4.79 | 209.68 | 0.29(-0.01-0.60) |
| Uganda | 533.79 | 3.08 | 1340.84 | 3.43 | 151.19 | 0.29(0.20-0.39) |
| France | 2492.93 | 4.32 | 2566.55 | 3.91 | 2.95 | -0.30(-0.36--0.24) |
| Austria | 462.5 | 5.96 | 510.63 | 5.81 | 10.41 | -0.30(-0.43--0.17) |
| Germany | 3777.4 | 4.72 | 4963.55 | 5.96 | 31.4 | 0.31(0.09-0.53) |
| Madagascar | 373.96 | 3.13 | 896.54 | 3.43 | 139.74 | 0.31(0.21-0.40) |
| Guinea | 207.32 | 3.37 | 434.12 | 3.67 | 109.4 | 0.32(0.26-0.38) |
| Moldova | 676.5 | 15.16 | 501.33 | 13.47 | -25.89 | 0.34(-0.01-0.69) |
| Japan | 3060.99 | 2.43 | 2736.47 | 2.13 | -10.6 | -0.37(-0.57--0.16) |
| Democratic Republic of the Congo | 1010.47 | 2.64 | 2437.35 | 3.01 | 141.21 | 0.39(0.30-0.48) |
| Spain | 1940.94 | 4.89 | 2102.01 | 4.53 | 8.3 | -0.41(-0.57--0.25) |
| Liberia | 83.12 | 4.19 | 221.49 | 4.69 | 166.47 | 0.46(0.23-0.68) |
| Puerto Rico | 417.84 | 11.57 | 498.49 | 13.6 | 19.3 | 0.46(0.38-0.53) |
| Mozambique | 440.59 | 3.06 | 1136.65 | 3.78 | 157.98 | 0.51(0.30-0.71) |
| New Zealand | 74.5 | 2.18 | 117.18 | 2.63 | 57.29 | 0.53(0.48-0.59) |
| Burundi | 222.91 | 4.05 | 414.65 | 3.8 | 86.02 | -0.54(-0.67--0.40) |
| Andorra | 1.96 | 3.61 | 3.52 | 4.41 | 79.59 | 0.56(0.41-0.72) |
| Cameroon | 413.54 | 3.99 | 1318.48 | 4.75 | 218.83 | 0.56(0.49-0.64) |
| Central African Republic | 72.96 | 2.67 | 149.02 | 3.22 | 104.25 | 0.58(0.42-0.74) |
| Chile | 931.29 | 7.01 | 1475.29 | 8.23 | 58.41 | 0.62(0.38-0.86) |
| Tanzania | 855.87 | 3.31 | 2226.11 | 4.12 | 160.1 | 0.62(0.48-0.77) |
| Egypt | 5392.56 | 9.81 | 10683.54 | 11.07 | 98.12 | 0.65(0.43-0.87) |
| Niger | 214.43 | 2.67 | 663.1 | 3.1 | 209.24 | 0.66(0.54-0.78) |
| Switzerland | 184.95 | 2.65 | 283.7 | 3.3 | 53.39 | 0.66(0.58-0.74) |
| Northern Mariana Islands | 1.58 | 3.49 | 1.97 | 4.39 | 24.68 | 0.67(0.57-0.76) |
| Greece | 360.5 | 3.46 | 444.79 | 4.28 | 23.38 | 0.68(0.57-0.80) |
| Iraq | 676.66 | 3.88 | 2059.26 | 4.76 | 204.33 | 0.74(0.52-0.97) |
| Netherlands | 411.01 | 2.76 | 648.12 | 3.81 | 57.69 | 0.77(0.62-0.92) |
| Kenya | 962.75 | 4.15 | 2489.8 | 5.15 | 158.61 | 0.77(0.68-0.85) |
| Italy | 2448.15 | 4.31 | 2119.18 | 3.5 | -13.44 | -0.77(-0.96--0.59) |
| Eritrea | 91.73 | 3.17 | 237.15 | 4.05 | 158.53 | 0.80(0.75-0.84) |
| Somalia | 240.92 | 3.36 | 683.1 | 4.05 | 183.54 | 0.84(0.67-1.00) |
| Australia | 401.18 | 2.38 | 697.17 | 2.91 | 73.78 | 0.85(0.70-1.01) |
| Angola | 298.8 | 2.92 | 1036.93 | 3.68 | 247.03 | 0.86(0.76-0.96) |
| Pakistan | 1635.42 | 1.51 | 4089.28 | 1.91 | 150.04 | 0.87(0.85-0.90) |
| The Bahamas | 18.16 | 7.05 | 36.52 | 9.73 | 101.1 | 0.92(0.82-1.02) |
| Belgium | 213.77 | 2.14 | 310.93 | 2.75 | 45.45 | 0.93(0.84-1.03) |
| Norway | 102.21 | 2.41 | 165.74 | 3.15 | 62.16 | 0.95(0.89-1.00) |
| Hungary | 904.23 | 8.65 | 609.17 | 6.26 | -32.63 | -0.95(-1.27--0.63) |
| China | 38615.2 | 3.23 | 63430.58 | 4.49 | 64.26 | 0.97(0.73-1.21) |
| Indonesia | 7402.06 | 3.98 | 13240.58 | 5.13 | 78.88 | 1.01(0.90-1.12) |
| Gabon | 35.71 | 3.64 | 80.48 | 4.73 | 125.37 | 1.02(0.95-1.08) |
| Mauritania | 71.55 | 3.45 | 184.05 | 4.7 | 157.23 | 1.03(0.92-1.15) |
| Bermuda | 4.75 | 8.01 | 7.37 | 11.19 | 55.16 | 1.06(0.87-1.25) |
| Serbia | 303.81 | 3.23 | 376.25 | 4.24 | 23.84 | 1.08(0.88-1.27) |
| Taiwan (Province of China) | 1050.35 | 5.15 | 1470.95 | 6.24 | 40.04 | 1.08(0.88-1.28) |
| Burkina Faso | 281 | 2.94 | 856.48 | 4.05 | 204.8 | 1.08(0.90-1.25) |
| Papua New Guinea | 87.6 | 2.16 | 265.92 | 2.88 | 203.56 | 1.08(1.00-1.16) |
| Guinea-Bissau | 34.2 | 3.39 | 81.47 | 4.39 | 138.22 | 1.13(0.97-1.29) |
| Barbados | 18.08 | 7.12 | 31.72 | 10.72 | 75.44 | 1.14(0.99-1.28) |
| Slovakia | 333.55 | 6.32 | 434.66 | 8.02 | 30.31 | 1.15(0.99-1.31) |
| Congo | 69.74 | 2.87 | 190.35 | 3.87 | 172.94 | 1.16(1.13-1.20) |
| Portugal | 583.17 | 5.76 | 463.76 | 4.34 | -20.48 | -1.16(-1.25--1.06) |
| Zimbabwe | 241.66 | 2.33 | 454.43 | 3.09 | 88.05 | 1.18(1.12-1.24) |
| Swaziland | 21.67 | 2.68 | 43.3 | 3.85 | 99.82 | 1.20(1.05-1.34) |
| Sao Tome and Principe | 5.2 | 4.26 | 11.61 | 5.8 | 123.27 | 1.20(1.05-1.35) |
| Czech Republic | 378.87 | 3.69 | 500.58 | 4.73 | 32.12 | 1.22(1.12-1.32) |
| Cyprus | 20.17 | 2.6 | 46.62 | 3.69 | 131.14 | 1.25(1.15-1.36) |
| Benin | 144.27 | 2.98 | 482.39 | 4.16 | 234.37 | 1.28(1.22-1.35) |
| Dominican Republic | 459.44 | 6.38 | 981.3 | 9.39 | 113.59 | 1.29(1.21-1.37) |
| Bulgaria | 358.28 | 4.02 | 409.54 | 5.81 | 14.31 | 1.30(1.19-1.40) |
| Qatar | 38.88 | 8.77 | 340.94 | 12.41 | 776.9 | 1.30(1.27-1.33) |
| Canada | 569.62 | 2.09 | 1066.33 | 2.96 | 87.2 | 1.31(1.28-1.34) |
| Guam | 4.51 | 3.29 | 8.04 | 4.79 | 78.27 | 1.32(1.27-1.37) |
| Singapore | 21.57 | 0.68 | 57.72 | 1.04 | 167.59 | 1.33(1.15-1.51) |
| The Gambia | 27.94 | 2.83 | 87.68 | 4.11 | 213.82 | 1.34(1.25-1.42) |
| Israel | 146.33 | 2.95 | 389.38 | 4.35 | 166.1 | 1.38(1.29-1.47) |
| Togo | 100.32 | 2.72 | 293.79 | 3.91 | 192.85 | 1.39(1.29-1.50) |
| Virgin Islands, U.S. | 9.12 | 8.59 | 13.21 | 12.59 | 44.85 | 1.39(1.30-1.48) |
| Malta | 9.75 | 2.64 | 16.24 | 3.74 | 66.56 | 1.40(1.28-1.51) |
| Vanuatu | 3.87 | 2.57 | 10.34 | 3.6 | 167.18 | 1.40(1.33-1.47) |
| Sudan | 871.81 | 4.31 | 2545.46 | 6.32 | 191.97 | 1.41(1.24-1.58) |
| Senegal | 199.73 | 2.62 | 594.61 | 4.05 | 197.71 | 1.42(1.23-1.60) |
| Antigua and Barbuda | 4.19 | 6.99 | 9.69 | 10.89 | 131.26 | 1.43(1.26-1.59) |
| Ghana | 441.79 | 2.96 | 1396.95 | 4.62 | 216.2 | 1.43(1.34-1.52) |
| Bhutan | 9.23 | 1.64 | 23.17 | 2.42 | 151.03 | 1.44(1.37-1.51) |
| Guyana | 61.78 | 7.93 | 81.44 | 10.97 | 31.82 | 1.48(1.35-1.62) |
| Bosnia and Herzegovina | 192.9 | 4.28 | 213.93 | 6.29 | 10.9 | 1.49(1.33-1.64) |
| Denmark | 141.62 | 2.76 | 246.06 | 4.29 | 73.75 | 1.50(1.20-1.80) |
| Cote d'Ivoire | 351.1 | 2.87 | 1039.46 | 4.16 | 196.06 | 1.50(1.44-1.57) |
| Sri Lanka | 495.29 | 2.88 | 1110.62 | 5.14 | 124.24 | 1.51(1.25-1.78) |
| Laos | 118.54 | 2.87 | 306.92 | 4.4 | 158.92 | 1.53(1.33-1.72) |
| Romania | 1480.15 | 6.33 | 1654.92 | 8.52 | 11.81 | 1.56(1.21-1.91) |
| Nepal | 224.26 | 1.16 | 537.73 | 1.8 | 139.78 | 1.57(1.34-1.79) |
| Georgia | 226.44 | 4.11 | 238.41 | 6.46 | 5.29 | 1.60(1.27-1.94) |
| Tajikistan | 134.75 | 2.51 | 366.4 | 3.96 | 171.91 | 1.60(1.47-1.74) |
| Comoros | 14.84 | 3.21 | 36.22 | 5.04 | 144.07 | 1.60(1.49-1.71) |
| India | 9956.88 | 1.14 | 25213.72 | 1.83 | 153.23 | 1.61(1.44-1.78) |
| Yemen | 432.01 | 3.15 | 1458.76 | 4.79 | 237.67 | 1.63(1.43-1.84) |
| Suriname | 27.02 | 6.96 | 63.58 | 11.11 | 135.31 | 1.65(1.56-1.74) |
| United States | 6428.31 | 2.54 | 13024.73 | 4.01 | 102.62 | 1.65(1.59-1.71) |
| Montenegro | 17.21 | 2.75 | 28.62 | 4.57 | 66.3 | 1.67(1.27-2.08) |
| Samoa | 4.68 | 2.85 | 8.74 | 4.4 | 86.75 | 1.69(1.56-1.82) |
| Lesotho | 42.13 | 2.33 | 72.59 | 3.73 | 72.3 | 1.70(1.65-1.75) |
| Namibia | 22.81 | 1.61 | 60.81 | 2.58 | 166.59 | 1.71(1.69-1.73) |
| Kyrgyzstan | 180.83 | 4.05 | 369.14 | 5.8 | 104.14 | 1.72(1.44-2.01) |
| Mexico | 10855.4 | 12.71 | 24901.96 | 19.67 | 129.4 | 1.72(1.62-1.83) |
| Brazil | 9811.11 | 6.57 | 22123.61 | 10.44 | 125.5 | 1.72(1.67-1.76) |
| Kiribati | 2.27 | 3.05 | 5.65 | 4.77 | 148.9 | 1.72(1.68-1.77) |
| Haiti | 305 | 4.78 | 906.81 | 7.67 | 197.31 | 1.74(1.64-1.85) |
| Myanmar | 1620.47 | 4.01 | 3380.3 | 6.4 | 108.6 | 1.75(1.73-1.77) |
| American Samoa | 1.6 | 3.29 | 3.02 | 5.42 | 88.75 | 1.77(1.71-1.82) |
| Mauritius | 41.13 | 3.74 | 82.44 | 6.48 | 100.44 | 1.79(1.62-1.96) |
| Uruguay | 113.24 | 3.61 | 208.37 | 6.09 | 84.01 | 1.79(1.63-1.94) |
| Cambodia | 390.13 | 3.74 | 943.4 | 5.85 | 141.82 | 1.81(1.64-1.98) |
| Sweden | 200.93 | 2.34 | 401.92 | 4 | 100.03 | 1.82(1.73-1.92) |
| Dominica | 3.85 | 5.21 | 6.58 | 9.55 | 70.91 | 1.87(1.73-2.01) |
| Azerbaijan | 265.26 | 3.62 | 637.69 | 6.24 | 140.4 | 1.87(1.80-1.95) |
| Argentina | 1141.75 | 3.45 | 2546.08 | 5.75 | 123 | 1.93(1.74-2.13) |
| Saint Lucia | 8.78 | 6.42 | 19.98 | 11.32 | 127.56 | 1.94(1.83-2.05) |
| Greenland | 0.91 | 1.64 | 1.55 | 2.76 | 70.33 | 1.96(1.85-2.07) |
| Bahrain | 29.75 | 5.86 | 148.47 | 10.1 | 399.06 | 1.97(1.72-2.21) |
| Grenada | 5.01 | 5.77 | 10.9 | 9.83 | 117.56 | 1.97(1.76-2.17) |
| Mongolia | 85.8 | 3.99 | 197.08 | 6.06 | 129.7 | 1.98(1.78-2.18) |
| Palestine | 71.04 | 3.49 | 279.13 | 5.75 | 292.92 | 1.98(1.82-2.14) |
| Solomon Islands | 6.92 | 2.05 | 21.44 | 3.36 | 209.83 | 2.00(1.85-2.14) |
| Equatorial Guinea | 12.04 | 2.84 | 60.54 | 4.5 | 402.82 | 2.01(1.77-2.26) |
| Timor-Leste | 16.13 | 2.06 | 45.88 | 3.56 | 184.44 | 2.01(1.93-2.08) |
| Bangladesh | 1419.63 | 1.3 | 3240.94 | 2.06 | 128.29 | 2.03(1.94-2.13) |
| North Korea | 441.36 | 2.17 | 1013.43 | 3.94 | 129.62 | 2.06(1.97-2.16) |
| Tonga | 3.21 | 3.33 | 5.95 | 5.79 | 85.36 | 2.10(1.85-2.35) |
| Federated States of Micronesia | 2.69 | 2.6 | 4.61 | 4.44 | 71.38 | 2.11(2.02-2.19) |
| Brunei | 3.85 | 1.49 | 12.16 | 2.81 | 215.84 | 2.15(1.98-2.32) |
| Panama | 113.81 | 4.77 | 360.02 | 9.18 | 216.33 | 2.18(2.04-2.31) |
| Trinidad and Tobago | 76.66 | 6.35 | 157.9 | 11.34 | 105.97 | 2.19(1.95-2.44) |
| Turkey | 1855.74 | 3.22 | 4998.09 | 6.21 | 169.33 | 2.25(1.93-2.57) |
| Venezuela | 1209.7 | 6.44 | 4044.06 | 13.12 | 234.3 | 2.26(1.93-2.60) |
| Botswana | 25.46 | 1.94 | 84.25 | 3.69 | 230.91 | 2.27(2.14-2.39) |
| South Korea | 2970.12 | 6.71 | 2256.32 | 4.28 | -24.03 | -2.27(-2.50--2.03) |
| Macedonia | 61.41 | 3.05 | 128.67 | 5.92 | 109.53 | 2.32(1.99-2.65) |
| Uzbekistan | 750.65 | 3.58 | 2049.81 | 6.36 | 173.07 | 2.32(2.13-2.50) |
| Paraguay | 172.88 | 4.29 | 570.22 | 8.23 | 229.84 | 2.36(2.24-2.47) |
| Guatemala | 663.76 | 8.29 | 2702.67 | 15.97 | 307.18 | 2.36(2.27-2.46) |
| Marshall Islands | 1.21 | 2.63 | 2.74 | 4.87 | 126.45 | 2.38(2.32-2.44) |
| United Kingdom | 1190.79 | 2.07 | 2688 | 4.03 | 125.73 | 2.40(2.18-2.62) |
| Belize | 9.6 | 5.09 | 40.58 | 10.27 | 322.71 | 2.41(2.28-2.54) |
| Cuba | 580.88 | 5.36 | 1205.91 | 10.6 | 107.6 | 2.42(2.37-2.47) |
| Honduras | 389.94 | 8.28 | 1470.47 | 15.48 | 277.1 | 2.43(2.33-2.53) |
| Albania | 89.5 | 2.71 | 145.27 | 5.25 | 62.31 | 2.46(2.23-2.70) |
| Jamaica | 93.79 | 3.95 | 237.73 | 8.55 | 153.47 | 2.52(2.23-2.81) |
| Iceland | 10.11 | 3.99 | 27.45 | 8.13 | 171.51 | 2.52(2.27-2.76) |
| Jordan | 149.35 | 3.99 | 883.75 | 8.3 | 491.73 | 2.57(2.35-2.79) |
| Syria | 443.28 | 3.49 | 1317.06 | 7.26 | 197.12 | 2.59(2.23-2.95) |
| Colombia | 1295.82 | 3.97 | 4445.68 | 8.78 | 243.08 | 2.60(2.41-2.79) |
| Morocco | 1041.85 | 4.13 | 2978.25 | 8.39 | 185.86 | 2.62(2.36-2.87) |
| Costa Rica | 187.14 | 6.15 | 632.52 | 13.59 | 237.99 | 2.62(2.51-2.74) |
| Cape Verde | 8.67 | 2.46 | 28.49 | 5.22 | 228.6 | 2.65(2.40-2.89) |
| Poland | 1246.82 | 3.19 | 2295.66 | 5.98 | 84.12 | 2.66(2.55-2.77) |
| Estonia | 45.15 | 2.88 | 69.78 | 5.31 | 54.55 | 2.68(2.46-2.91) |
| Peru | 1274.25 | 5.82 | 3871.71 | 11.65 | 203.84 | 2.71(2.59-2.83) |
| Djibouti | 13.86 | 2.78 | 62.88 | 5.65 | 353.68 | 2.72(2.55-2.88) |
| Fiji | 12.08 | 1.58 | 31.34 | 3.46 | 159.44 | 2.72(2.56-2.89) |
| Latvia | 78.35 | 2.88 | 104.07 | 5.35 | 32.83 | 2.78(2.60-2.95) |
| Turkmenistan | 138.68 | 3.75 | 363.5 | 7.3 | 162.11 | 2.78(2.65-2.92) |
| Lebanon | 158.05 | 3.85 | 660.38 | 7.76 | 317.83 | 2.82(2.59-3.05) |
| Seychelles | 2.9 | 3.97 | 8.63 | 8.55 | 197.59 | 2.86(2.75-2.96) |
| Finland | 137.72 | 2.75 | 304.19 | 5.51 | 120.88 | 2.88(2.64-3.11) |
| Tunisia | 545.41 | 6.48 | 1592.68 | 13.92 | 192.02 | 2.92(2.75-3.08) |
| Vietnam | 2163.05 | 3.18 | 6683.78 | 6.95 | 209 | 2.94(2.64-3.24) |
| Bolivia | 394.43 | 6.11 | 1505.97 | 13.05 | 281.81 | 2.98(2.91-3.05) |
| Ireland | 63.32 | 1.76 | 186.66 | 3.84 | 194.79 | 3.00(2.85-3.14) |
| El Salvador | 388.63 | 7.41 | 978.2 | 16.07 | 151.7 | 3.03(2.98-3.08) |
| Saint Vincent and the Grenadines | 5.85 | 5.31 | 14.15 | 12.4 | 141.88 | 3.04(2.94-3.14) |
| Thailand | 2126.28 | 3.73 | 6095.63 | 8.63 | 186.68 | 3.05(2.92-3.19) |
| Malaysia | 326.28 | 1.85 | 1327.61 | 4.33 | 306.89 | 3.06(2.81-3.30) |
| Algeria | 881.34 | 3.46 | 3206.49 | 7.92 | 263.82 | 3.17(2.95-3.40) |
| Saudi Arabia | 746.81 | 4.56 | 3625.34 | 10.53 | 385.44 | 3.20(3.12-3.29) |
| Ecuador | 673.47 | 6.72 | 2585.84 | 15.5 | 283.96 | 3.26(3.18-3.34) |
| United Arab Emirates | 101.66 | 5.38 | 1239.45 | 12.73 | 1119.21 | 3.29(2.97-3.62) |
| Libya | 200.16 | 4.78 | 750.28 | 10.86 | 274.84 | 3.35(3.15-3.55) |
| Kuwait | 69.95 | 3.94 | 411.17 | 9.65 | 487.81 | 3.38(3.23-3.53) |
| Russian Federation | 5497.82 | 3.63 | 10993.5 | 7.52 | 99.96 | 3.43(3.16-3.70) |
| Ukraine | 1994.49 | 3.79 | 3545.17 | 7.93 | 77.75 | 3.47(3.14-3.81) |
| Lithuania | 113.05 | 3.01 | 180.92 | 6.35 | 60.04 | 3.52(3.18-3.86) |
| Kazakhstan | 474.69 | 2.82 | 1129.2 | 6.31 | 137.88 | 3.52(3.29-3.75) |
| Nicaragua | 243.85 | 6.26 | 976.31 | 15.26 | 300.37 | 3.54(3.48-3.61) |
| Maldives | 3.44 | 1.57 | 19.21 | 4.19 | 458.43 | 3.69(3.20-4.19) |
| Philippines | 648.6 | 1.02 | 2937.02 | 2.84 | 352.82 | 3.69(3.59-3.80) |
| Armenia | 88.97 | 2.6 | 192.66 | 6.36 | 116.54 | 3.71(3.52-3.91) |
| Oman | 67.89 | 3.54 | 434.95 | 9.59 | 540.67 | 3.87(3.39-4.35) |
| Belarus | 285.42 | 2.73 | 568.09 | 5.99 | 99.04 | 3.91(3.55-4.26) |
| Iran | 1143.88 | 1.98 | 4085.84 | 4.97 | 257.19 | 3.94(3.71-4.18) |
